# Chemotherapeutic resistance of head and neck squamous cell carcinoma is mediated by EpCAM induction driven by IL-6/p62 associated Nrf2-antioxidant pathway activation

**DOI:** 10.1038/s41419-020-02907-x

**Published:** 2020-08-20

**Authors:** Abu Shadat M. Noman, Rashed R. Parag, Muhammad I. Rashid, Shafiqul Islam, Mohammad Z. Rahman, Ali A. Chowdhury, Afrin Sultana, Chandsultana Jerin, Ayesha Siddiqua, Lutfur Rahman, Junayed Nayeem, Sonam Akther, Sunanda Baidya, Rajib K. Shil, Mizanur Rahman, Afsana Shirin, Reaz Mahmud, S. M. Ikram Hossain, Sharmin A. Sumi, Arfina Chowdhury, Shabnam B. Basher, Abul Hasan, Shammy Bithy, Jannatul Aklima, Nabila Chowdhury, Muhammad N. Hasan, Tahmina Banu, Srikanta Chowdhury, Muhammad M. Hossain, Herman Yeger, Walid A. Farhat, Syed S. Islam

**Affiliations:** 1 Department of Biochemistry and Molecular Biology, University of Chittagong, Chittagong, Bangladesh; 2 Department of Pathology, McGill University, Montreal, QC Canada; 3 Department of Pathology, Chittagong Medical College Hospital, Chittagong, Bangladesh; 4 Department of Radiotherapy, Chittagong Medical College Hospital, Chittagong, Bangladesh; 5 Department of Biochemistry, Rangamati Medical College, Rangamati, Bangladesh; 6 Chittagong Research Institute for Children Surgery (CRICS), Chittagong, Bangladesh; 7 Developmental and Stem Cell Biology, Peter Gilgan Centre for Research and Learning, The Hospital for Sick Children, Toronto, ON Canada; 8 Division of Pediatric Urology, American Family Children’s Hospital, University of Wisconsin School of Medicine and Public Health, Madison, WI USA; 9 Department of Molecular Oncology, King Faisal Specialist Hospital and Research Centre, Riyadh, Saudi Arabia; 10 School of Medicine, Al-Faisal University, Riyadh, Saudi Arabia

**Keywords:** Oral cancer, Oral cancer

## Abstract

Overexpression of epithelial cell adhesion molecule (EpCAM) has been associated with chemotherapeutic resistance, leads to aggressive tumor behavior, and results in an adverse clinical outcome. The molecular mechanism by which EpCAM enrichment is linked to therapeutic resistance via Nrf2, a key regulator of antioxidant genes is unknown. We have investigated the link between EpCAM and the Nrf2 pathway in light of therapeutic resistance using head and neck squamous cell carcinoma (HNSCC) patient tumor samples and cell lines. We report that EpCAM was highly expressed in Nrf2-positive and HPV-negative HNSCC cells. In addition, cisplatin-resistant tumor cells consisted of a higher proportion of EpCAM^high^ cells compared to the cisplatin sensitive counterpart. EpCAM^high^ populations exhibited resistance to cisplatin, a higher efficiency in colony formation, sphere growth and invasion capacity, and demonstrated reduced reactive oxygen species (ROS) activity. Furthermore, Nrf2 expression was significantly higher in EpCAM^high^ populations. Mechanistically, expression of Nrf2 and its target genes were most prominently observed in EpCAM^high^ populations. Silencing of EpCAM expression resulted in the attenuation of expressions of Nrf2 and SOD1 concomitant with a reduction of Sox2 expression. On the other hand, silencing of Nrf2 expression rendered EpCAM^high^ populations sensitive to cisplatin treatment accompanied by the inhibition of colony formation, sphere formation, and invasion efficiency and increased ROS activity. The molecular mechanistic link between EpCAM expression and activation of Nrf2 was found to be a concerted interaction of interleukin-6 (IL-6) and p62. Silencing of p62 expression in EpCAM^high^ populations resulted in the attenuation of Nrf2 pathway activation suggesting that Nrf2 pathway activation promoted resistance to cisplatin in EpCAM^high^ populations. We propose that therapeutic targeting the Nrf2-EpCAM axis might be an excellent approach to modulate stress resistance and thereby survival of HNSCC patients enriched in EpCAM^high^ populations.

## Introduction

Head and neck squamous cell carcinoma (HNSCC) affects more than 800,000 patients per year^1,2^. Resistance to chemotherapeutic drugs limits the overall treatment outcome in HNSCC patients^3^. Response to chemotherapeutic drugs is partly mediated by the Keap1-Nrf2 signaling system^4^. Nrf2/NFE2L2 (Nuclear factor, erythroid 2-like 2) is a key transcription factor, which in the normal basal state functions as cytoprotective response to oxidative and electrophilic stress. Under oxidative stress state, Nrf2 dissociates from cytoplasmic inhibitor Keap1 (Kelch like ECH-associated protein 1), translocate into the nucleus, and activates Nrf2 transcriptional genes and protects cells against oxidative stress, mediates detoxification, and participates in ATP-dependent drug efflux^5^. Abnormalities of Keap1-Nrf2 pathway lead to a mechanism of oncogenesis and chemo- and radio-resistance in a variety of cancers including HNSCC^4^. Inhibition of Nrf2 expression by siRNA augmented carboplatin-induced tumor growth inhibition in a xenograft mouse model^6^. Recent studies have indicated that Keap1-Nrf2 pathway is engaged in sustaining CSC (cancer stem cell)-like properties in cancers and causes resistance to therapeutic agents.

CSCs exhibit enhanced self-renewal properties, lead to disease recurrence, and most importantly exhibit the strongest therapeutic resistance within the tumor cells population^7–9^. Elevated expression of Nrf2 target genes contribute to therapeutic resistance and facilitate survival of CSCs^10^. Several cell surface markers, such as CD44, CD133, CD24, CD49f, and ALDH have been proposed for the detection and isolation of CSCs from tumors^11,12^. Many studies also emphasized the potential use of epithelial cell adhesion molecule (EpCAM) as a marker of CSCs, due to its ubiquitous overexpression in tumors^13^. EpCAM was originally identified as a novel tumor-specific cell surface antigen and overexpressed in a large number of cancers^14–17^ and involved in cell migration, proliferation, and differentiation^18^. Due to its wide expression, EpCAM may be a potential target for molecular intervention for therapeutically resistant tumors and requires further investigation.

A recent study reported that Nrf2 knockdown inhibits the self-renewal capacity of glioma stem cells^19^. Furthermore, Nrf2 signaling is activated in spheroids in breast and colon cancer cells where high Nrf2 activity in spheroids has correlated with therapeutic resistance^20^. However, it is unknown how the Nrf2 pathway and EpCAM interact and play roles in the development of chemotherapeutic resistance. In view of the importance of EpCAM and Nrf2 signaling in the development of chemoresistance, and the limited understanding of the link between EpCAM and the Nrf2 pathway, we investigated the potential role of Nrf2 signaling in CSCs with special emphasis on EpCAM-enriched cells that leads to chemotherapeutic resistance.

## Results

### Cancer stem cell markers are upregulated in HPV-negative and Nrf2 overexpressing HNSCC tumors

**Fig. 1:**
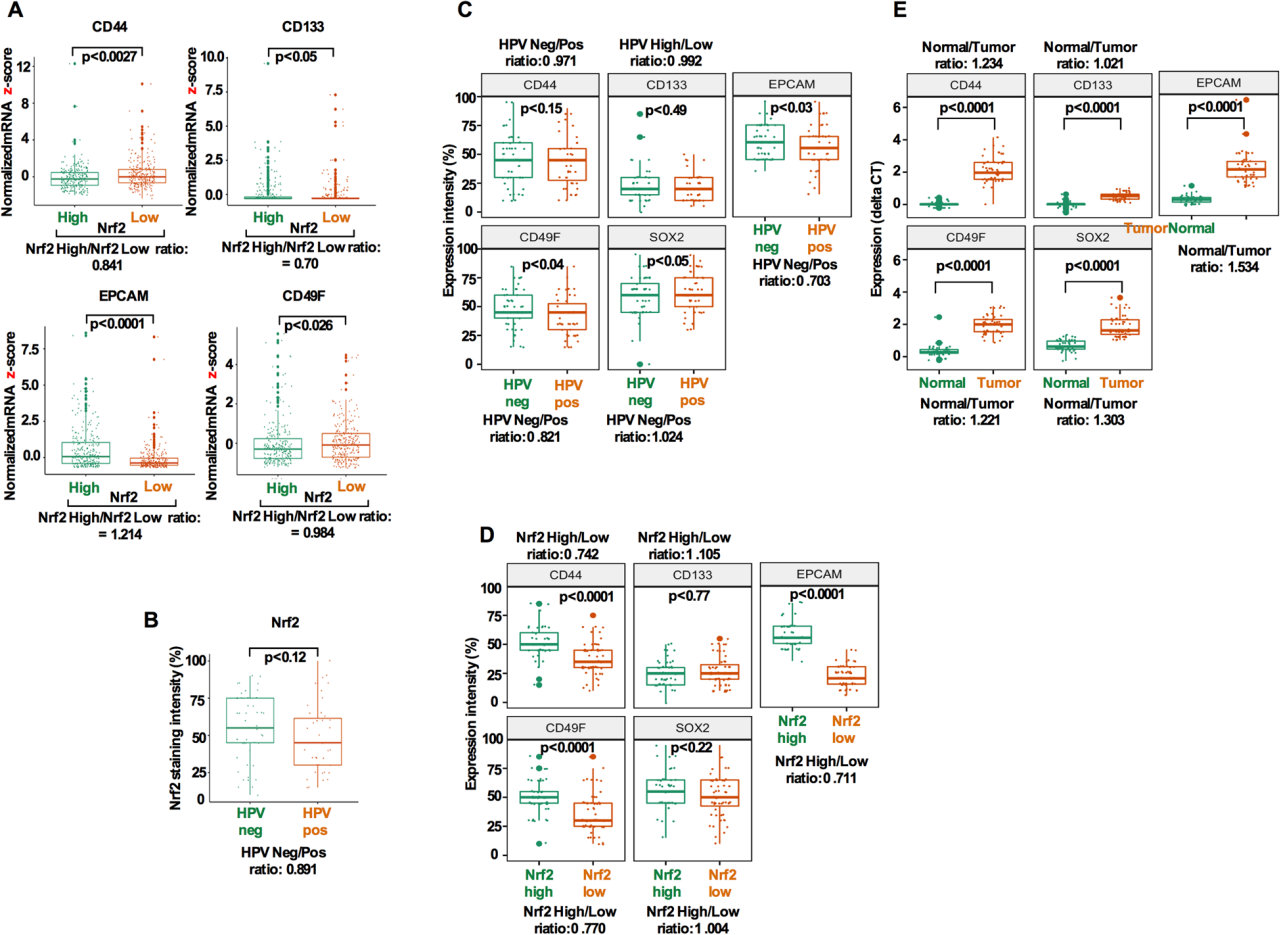
Cancer stem cell markers (CSCs) are upregulated in HPV-negative/Nrf2 overexpressing HNSCC tumors. **a** CD44, CD133, EpCAM, and CD49f were compared between Nrf2-high and -low groups using the TCGA dataset. Ratios were calculated by dividing the mRNA expression of the Nrf2-high group by that of the Nrf2-low group. **b** Nrf2 expression was compared between HPV(+) and HPV(−) group using our own dataset (*n* = 100). Ratio was calculated by dividing the expression intensity of the HPV(+) group by that of the HPV(−) group. **c** CSCs expression compared between HPV(+) and HPV(−) group. Ratio was calculated by dividing the expression intensity of the HPV(+) group by that of the HPV(−) group. **d** Expression of CSCs was compared between Nrf2-high and Nrf2-low group. Ratios were calculated by dividing the expression intensity of the Nrf2-high group by that of the Nrf2-low group. **e** Cancer stem markers were compared between HNSCC and matched normal tissues. Ratio was calculated by dividing the mRNA expression of the tumor sample by that of the matched normal samples. In all cases, whiskers indicate the maximum and the minimum values. *p*-values were calculated using Student’s *t* test.

Given the role of Nrf2 signaling in chemotherapeutic resistance and CSC survival^21,22^, we first explored the expression of several prominent CSC markers in HNSCC using 513 cases from TCGA dataset. We used normalized mRNA z-scores and compared several CSC markers within Nrf2-high and Nrf2-low tumors. Statistically significant differences were obtained for all CSC markers comparing the Nrf2-high and Nrf2-low groups with the most significant relationship found in EpCAM (*p* < 0.0001; Fig. 1a). Since, HPV (human papillomavirus) has emerged as a novel risk factor for HNSCCs^23^, we therefore compared Nrf2 expression in HPV-positive and HPV-negative patient groups from our own archived tumor samples. No significant differences were noted between the HPV groups and Nrf2 expression (*p* = 0.12; Fig. 1b). A significant expression difference was noted in EpCAM, CD49f, and stemness factor Sox2 (*p* = 0.03; *p* = 0.04; *p* = 0.05; Fig. 1c) in HPV-negative versus HPV-positive groups. In addition, CD44 (*p* < 0.0001), CD49f (*p* < 0.0001), EpCAM (*p* < 0.0001), and Sox2 (*p* = 0.02) showed significantly higher expressions in the Nrf2-high group (Fig. 1d). CSC markers including Sox2 were significantly increased in the tumor tissue compared with matched normal tissues (Fig. 1e).

### EpCAM is expressed in cisplatin resistant cells and EpCAM inhibition sensitizes cells to cisplatin and inhibits HNSCC cell proliferation

**Fig. 2:**
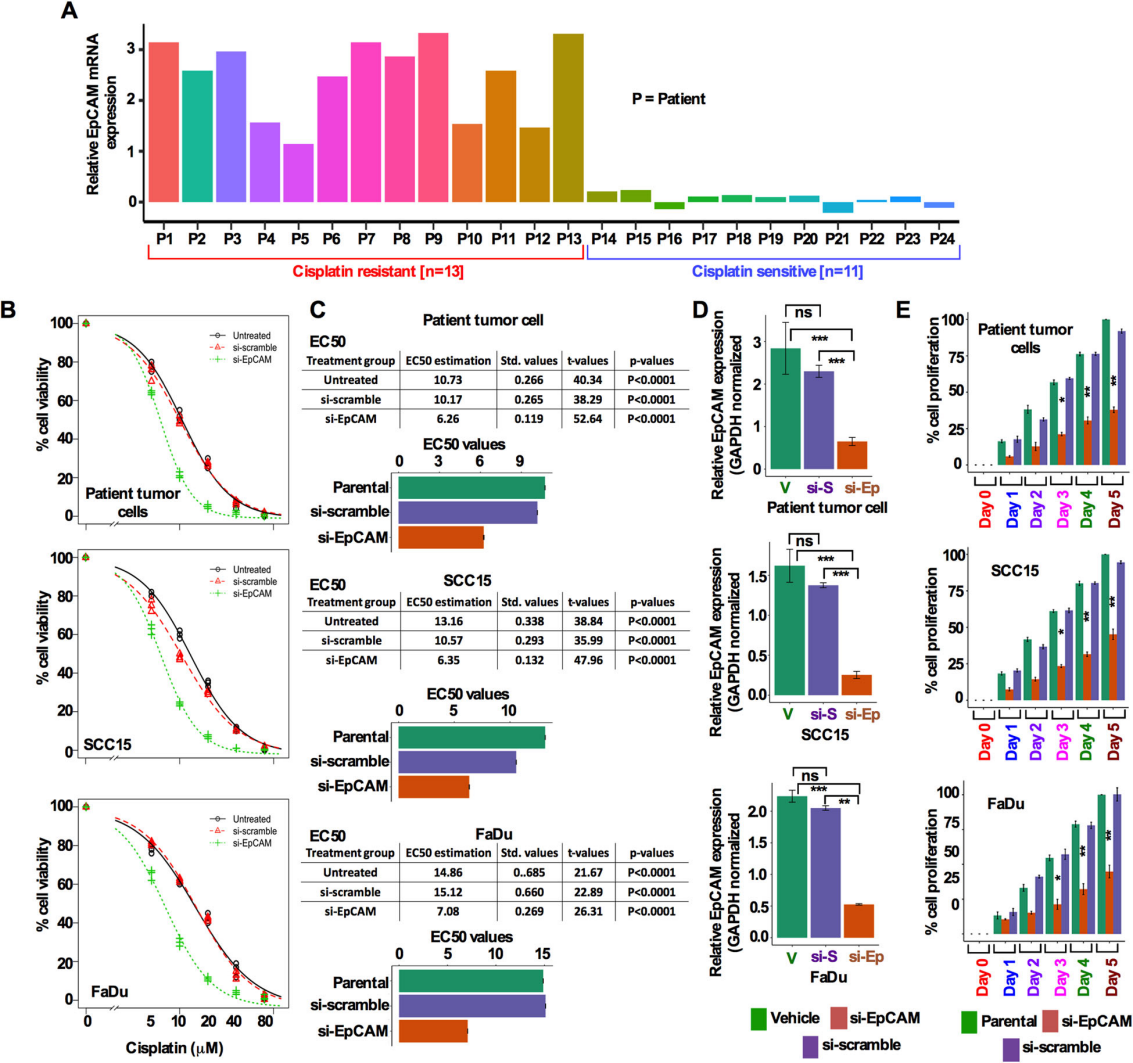
EpCAM is expressed in cisplatin resistant cells and EpCAM inhibition sensitizes HNSCC cells to cisplatin and inhibits HNSCC cell proliferation. **a** Expression of EpCAM mRNA in HNSCC patients’ cisplatin resistant and sensitive tumor cells. **b** Dose-response and cell viability of HNSCC patient tumor cells (top panel), SCC15 (middle panel), and FaDu (bottom panel) cells. Cell viability of siEpCAM and scrambled siRNA transfected cells were monitored following exposures of cells to different concentrations of cisplatin. **c** EC50 of cisplatin in parental, si-scrambled, and siEpCAM transfected patient tumor cells (top panel), SCC15 (middle panel), and FaDu (bottom panel) cells. The EC50 differences between si-scramble and siEpCAM cells were compared. **d** Relative EpCAM mRNA expression in HNSCC patient tumor cells (top panel), SCC15 (middle panel), and FaDu (bottom panel) cells following transfection of cells by si-scrambled and siEpCAM. *p*-values were calculated using Student’s *t* test. **e** Cell proliferation was determined following transfection of cells by si-scrambled and siEpCAM. Si-scrambled and siEpCAM cell growth was compared on day 5. ns denote not significant, *p < 0.05, **p < 0.01, ***p < 0.001.

Next, we evaluated EpCAM transcript levels from a group of cisplatin resistant (*n* = 13) and sensitive (*n* = 11) HNSCC patient tumors. Cisplatin resistant tumors (56.27%) showed relatively high expression of EpCAM compared with cisplatin sensitive (45.83%) tumors (Fig. 2a). This finding led us to test the cisplatin resistance in vitro, for which we established a line of cisplatin-resistant FaDu cells, termed as FaDuRes. We established a cisplatin-resistant FaDuRes cells by maintaining parental FaDu cells in a series of cisplatin concentrations for 2 weeks before these cells were stably grown in 5 μM cisplatin. As shown in Supplementary Fig. S1A, FaDuRes cells exhibited higher resistance to cisplatin treatment compared to parental FaDu cells. We then treated FaDu cells 5 μM of cisplatin for 5 days and analyzed the EpCAM expression by western blot. FaDu cells maintained in 5 μM of cisplatin showed higher EpCAM expression in contrast to untreated parental cells (Supplementary Fig. S1B). To assess the role of EpCAM in cisplatin resistance, untreated patient tumor cells, SCC15 and FaDu cells, were transfected with siEpCAM and si-scramble for 48 h, washed and followed by cisplatin treatment for an additional 48 h and assessed for cell viability (Supplementary Fig. S2). Whereas parental cells were found to be somewhat resistant to cisplatin treatment, knockdown of EpCAM with siEpCAM enhanced the sensitivity to cisplatin treatment (Fig. 2b). To examine the resistance further, cells were treated with different concentrations of cisplatin and determined the EC50 (Fig. 2c). Furthermore, silencing EpCAM significantly reduced EpCAM transcript level and inhibited cell proliferation (Fig. 2d, e).

### Chemotherapeutic resistance is associated with increased Nrf2 transcriptional activity and EpCAM overexpression

**Fig. 3:**
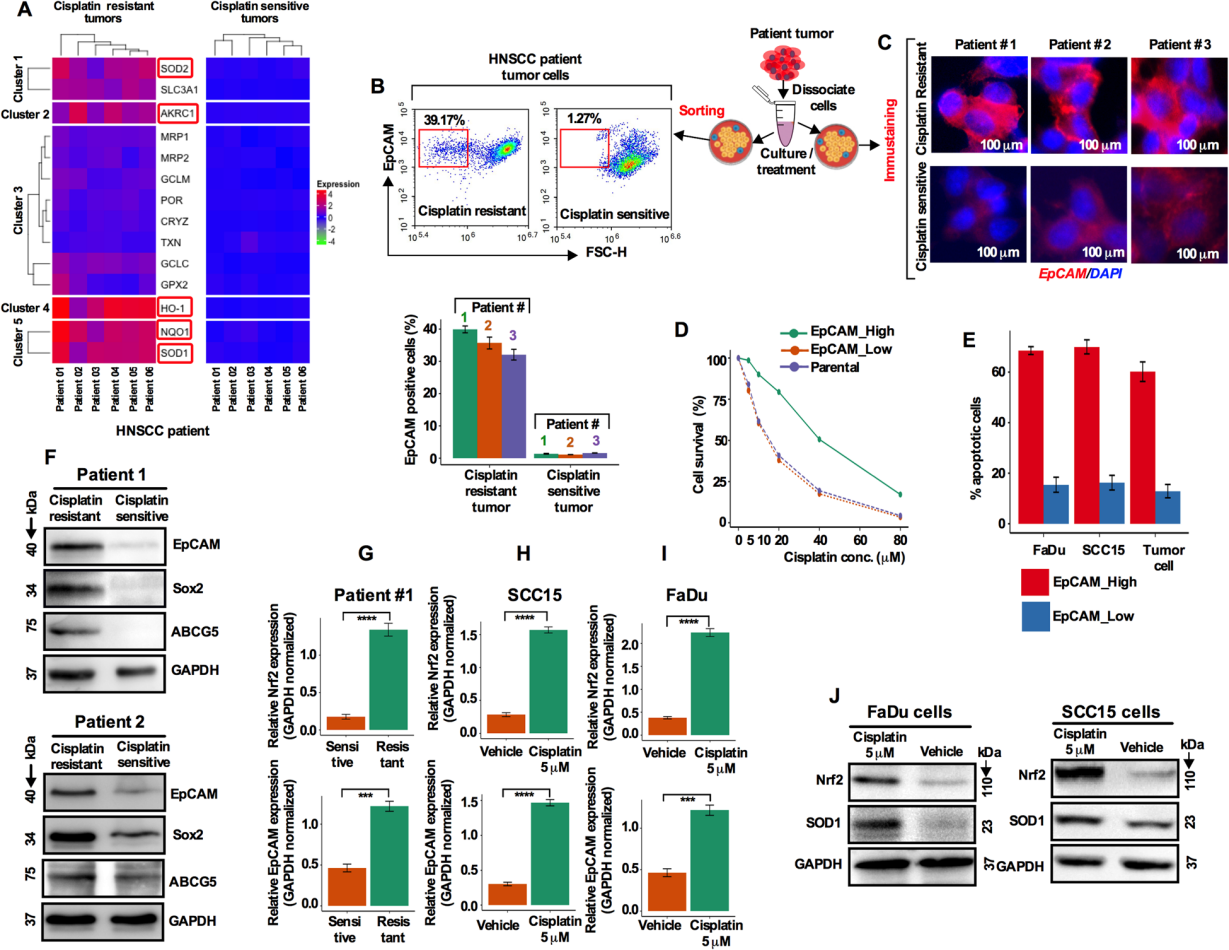
Chemotherapeutic resistance is associated with increased Nrf2 transcriptional activity and EpCAM overexpression. **a** Heat map of hierarchically clustering based on the expression of Nrf2 pathway target genes reveals distinct expressions in cisplatin resistant and sensitive patient tumor cells. Significantly upregulated gene expression intensity marked as red and downregulated genes are marked as blue. **b** HNSCC patient tumor cells were isolated from treatment resistant and sensitive patients and EpCAM positive cells identified by flow cytometry. **c** Immunofluorescence images of EpCAM expression were captured from the cultured cisplatin resistant and sensitive HNSCC patient tumor cells. Scale bar 100 µm. **d** Cell viability determination in parental, EpCAM^high^, and EpCAM^low^ cells after treating the cells with different cisplatin concentrations. **e** Apoptotic cell determination in EpCAM^high^ and EpCAM^low^ cells after cisplatin (10 µM) treatment. **f** EpCAM, Sox2, and ABCG5 protein levels were determined from cisplatin resistant and sensitive patient tumor cells by western blotting. **g** Transcript levels of EpCAM and Nrf2 in HNSCC patient tumor cells assessed by qRT-PCR. Values represents ±SD for three independent experiments. **h**–**i** Transcript levels of EpCAM and Nrf2 in SCC15 and FaDu cells assessed by qRT-PCR. Values represents ±SD for three independent experiments. **J** Nrf2 and SOD1 protein expression in cisplatin-treated SCC15 and FaDu cells.

It was reported that inhibition of Nrf2 reverses the resistance to cisplatin of HNSCC cells^22^. To further assess the role of Nrf2 in chemotherapeutic resistance, we compared Nrf2 target genes in cisplatin sensitive (*n* = 6) and resistant (*n* = 6) HNSCC patients’ tumor cells by real-time quantitative polymerase chain reaction (qRT-PCR). The unsupervised heat map analysis showed that Nrf2 target genes SOD2, SLC3A1, AKRC1, GCLC, HO-1, NQO1, and SOD1 were highly upregulated in the cisplatin-resistant tumor cells compared to the cisplatin sensitive tumor counterparts (Fig. 3a), suggesting that cisplatin treatment potentially plays a significant role in Nrf2 pathway activation. To establish the link between Nrf2 and EpCAM in resistance, freshly isolated cisplatin resistant (*n* = 3) and sensitive (*n* = 3) patient tumor cells were subjected to flow cytometry analysis and quantified the EpCAM expression. Approximately, 39.17% EpCAM-positive cell population was found in cisplatin resistant tumors while only a 1.27% EpCAM-positive cell population was detectable in cisplatin sensitive tumors (Fig. 3b). Based on these EpCAM cell fractions in resistant and sensitive groups we hereafter termed these two populations EpCAM^**high**^ and EpCAM^**low**^. Immunostaining for EpCAM in cisplatin resistance (*n* = 3) tissues showed enhanced expression of EpCAM (Fig. 3c). Fluorescence-activated cell sorting (FACS) sorted EpCAM^**high**^ cell fraction was highly resistant to cisplatin compared to the EpCAM^low^ cell fraction (Fig. 3d, e).

Functionally, tumors resistant to cisplatin showed enhanced expression of EpCAM coupled with the increased level of Sox2 and ABCG5 (Fig. 3f) indicating enrichment of EpCAM coincident with stem-like and drug resistant features in cells. We then analyzed Nrf2 and EpCAM transcript levels by qRT-PCR and found that both transcripts were increased in cisplatin-resistant tumor cells (Fig. 3g), suggesting that resistance to cisplatin is due in part to an increased level of Nrf2 transcriptional activity and EpCAM overexpression. To confirm this finding in cell lines, SCC15 and FaDu cells were treated with cisplatin (5 μM) for 5 days and were assessed for the level of Nrf2 and EpCAM transcripts. Cisplatin treatment significantly increased the Nrf2 and EpCAM expression levels (Fig. 3h–j). Immunoblot analysis confirmed that both Nrf2 and SOD1 expression were higher in cisplatin treated cells (Fig. 3j).

### Nrf2 pathway is predominantly activated in EpCAM^high^ cells and EpCAM knockdown inactivates the Nrf2-ARE pathway

**Fig. 4:**
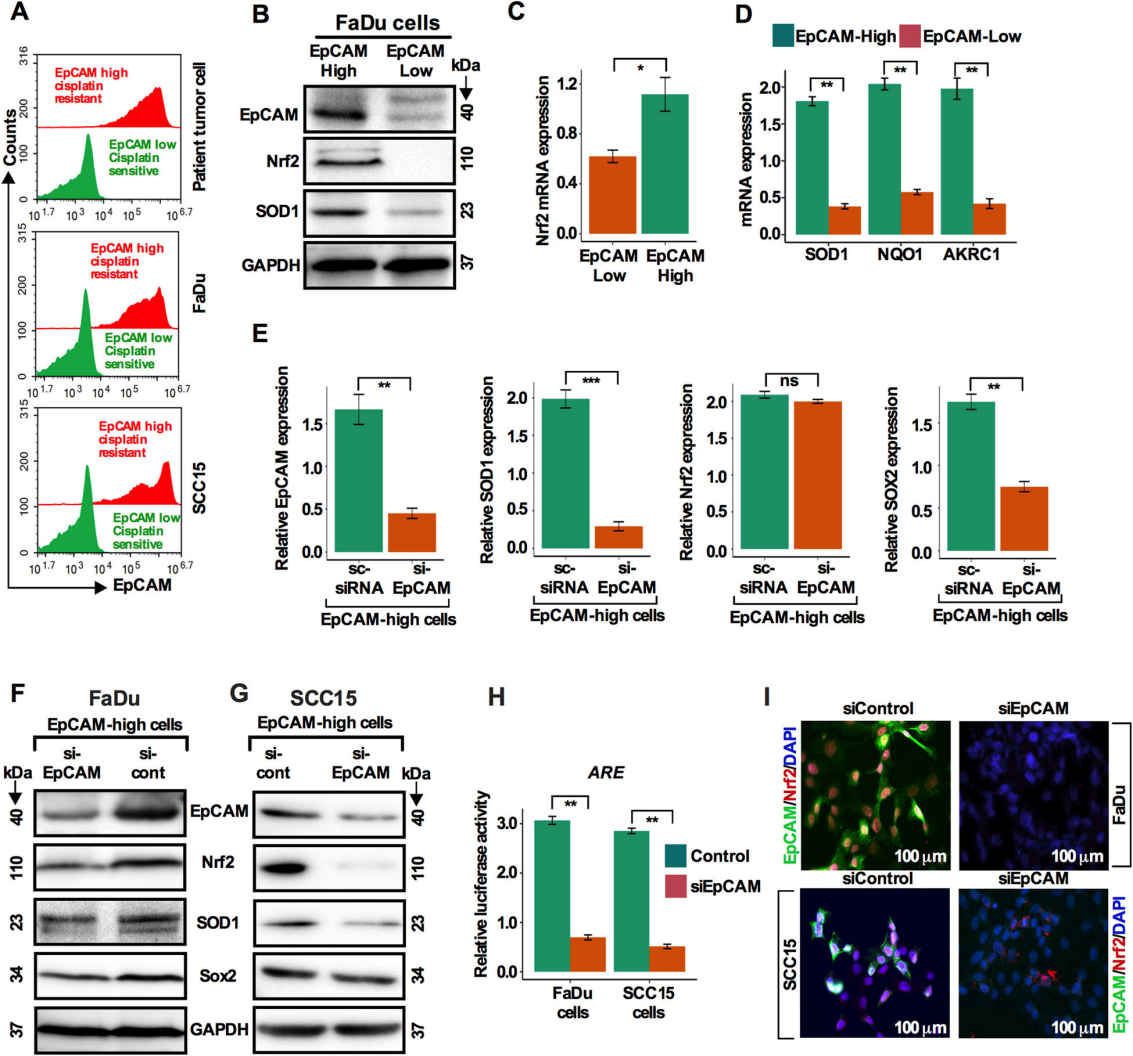
Nrf2 pathway is predominantly activated in EpCAM^high^ cells and EpCAM knockdown inactivates the Nrf2-ARE pathway. **a**. EpCAM^high^ and EpCAM^low^ cells were determined in HNSCC patient tumor cells, SCC15 and FaDu cells using flow cytometry. **b**. Total cellular protein levels of EpCAM, Nrf2, and SOD1 were determined in EpCAM^high^ and EpCAM^low^ cells by western blot analysis. **c**. Nrf2 transcript levels in EpCAM^high^ and EpCAM^low^ cells. *p < 0.05 compared to EpCAM^low^ group. **d** SOD1, NQO1, and AKRC1 transcript levels in EpCAM^high^ and EpCAM^low^cells. **p < 0.01 com*p*ared to EpCAM^low^ group. **e** FaDu cells were transfected with siEpCAM and scrambled siRNA and EpCAM, Nrf2, SOD1, and Sox2 transcript levels were determined by qRT-PCR analysis in EpCAM^high^ cells. **f**, **g** Protein levels of EpCAM, Nrf2, SOD1, and Sox2 were determined by western blot analysis in FaDu and SCC15 HNSCC cells after silencing EpCAM in EpCAM^high^ cells. **h** A Luciferase assay was used to detect reporter gene activity from AREs. **i** Immunostaining of HNSCC cells stained with EpCAM (green), Nrf2 (red), and DAPI (blue) after EpCAM knockdown. Scale bar: 100 μm.

To explore if the Nrf2 pathway is exclusively activated in EpCAM^high^ cells, freshly isolated cisplatin-resistant and sensitive patient tumor cells were FACS sorted. EpCAM^high^ cells were predominantly detected in the cisplatin-resistant cell fraction compared to sensitive cells (Fig. 4a). Cells were treated either with cisplatin or vehicle for 5 days and analyzed by flow cytometry. The results corroborated the results obtained in patient tumor cells (Fig. 4a). Next, we cultured cisplatin treated FaDu cells in growth supplemented CSC medium for 10 days. FACS sorted for EpCAM^high^ and EpCAM^low^ cells were re-cultured in CSC medium for an additional 10 days. EpCAM^high^ cells overexpressed EpCAM, Nrf2, and SOD1 proteins and Nrf2 transcripts (Fig. 4b, c). In addition, EpCAM^high^ cells overexpressed SOD1, NQO1, and AKRC1 transcripts (Fig. 4d). These results indicated that the Nrf2 signaling pathway is exclusively activated in the EpCAM^high^ cells. To determine whether the elevated Nrf2 level in EpCAM^high^ cells is EpCAM dependent, we silenced EpCAM expression by siEpCAM and observed that EpCAM, Nrf2, SOD1, and Sox2 proteins and transcripts were attenuated in EpCAM^high^ cells (Fig. 4e–g). These observations prompted us to hypothesize that EpCAM might regulate the expression of antioxidant factors via the Nrf2-ARE (antioxidant response elements) pathway. This hypothesis was tested by transfection of FaDu and SCC15 cells with an ARE luciferase reporter. HNSCC cells with or without EpCAM knockdown were transiently transfected with an ARE luciferase reporter plasmid. At 24 h post transfection, the cells were assayed for luciferase activity. EpCAM knockdown decreased the luciferase reporter activity with a comparable decreased staining intensity in EpCAM and Nrf2 (Fig. 4h, i). These results suggest that the inhibitory effects of EpCAM knockdown on cell growth and cisplatin resistance correlates with the degree of Nrf2 activation in CSC-like EpCAM^high^ cells.

### Nrf2 inhibition in EpCAM^high^ cells sensitizes cells to cisplatin treatment coupled with the abrogation of production of reactive oxygen species

**Fig. 5:**
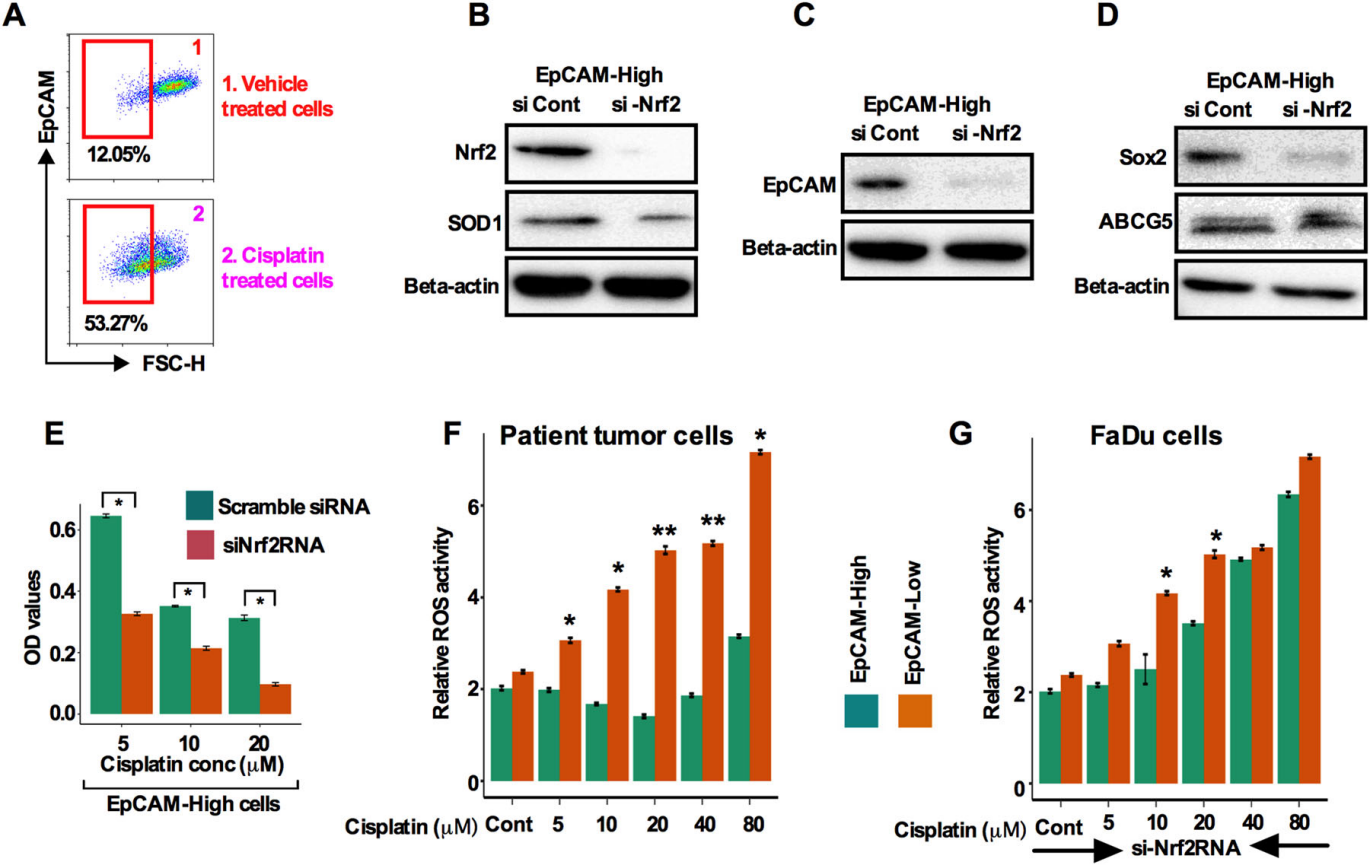
Nrf2 inhibition in EpCAM^high^ cells sensitizes cells to cisplatin treatment coupled with the abrogation of production of reactive oxygen species (ROS). **A**. EpCAM^high^ and EpCAM^low^ cells were determined in HNSCC patient tumor cisplatin-resistant and untreated tumor cells using flow cytometry (legend: 1-Untreated and, 2-Cisplatin resistant patient tumor cells). **B**. Protein levels of Nrf2 and SOD1 were measured in siNrf2 silenced and si-scrambled EpCAM^high^ cells by western blot. **c** EpCAM protein was measured in siNrf2 silenced and si-scramble cells by western blot. **d** Sox2 and ABCG5 protein levels were determined in siNrf2 silenced and si-scrambled EpCAM^high^ cells by western blot. **e** Cell viability was analyzed after incubation of cisplatin for 72 h in siNrf2 and si-scrambled EpCAM^high^ cells. **e**, **f** ROS activity was measured from (**e**) patient tumor cells and (**f**) FaDu cells FACS sorted EpCAM^high^ and EpCAM^low^ cells after cisplatin or siNrf2RNA treatment. Values represent ±SD from triplicate sampled wells. *p < 0.05 compared with untreated groups.

An increasing number of reports suggest that cisplatin-mediated CSC enrichment and resulting resistance substantially limits the positive outcome of the disease^24^. Furthermore, in a group of HNSCC patient tumors, high expression of EpCAM has been reported to correlate with therapeutic resistance^25,26^. To explore the possible functional link between chemotherapeutic resistance and EpCAM, we first sorted EpCAM^high^ cells by flow cytometry from cisplatin and vehicle-treated FaDu cells and found that higher percentage of EpCAM^high^ cells (53.27% vs 12.05%; Fig. 5a) in cisplatin treated cells. Knockdown of Nrf2 in EpCAM^high^ cells attenuated the expression of Nrf2 and Nrf2 target gene SOD1 proteins (Fig. 5b) concomitant with the attenuation in expression of EpCAM, Sox2, and ABCG5 (Fig. 5c, d). Additionally, Nrf2 silencing in EpCAM^high^ cells showed a significant increased sensitivity to cisplatin treatment (Fig. 5e).

Various antioxidant enzymes are induced by Nrf2 pathway activation that reduce the intracellular ROS level resulting in cells becoming drug resistant^27^. Hence, we speculated that chemotherapeutic resistance might likely be due to the reduction of ROS in EpCAM^high^ cells. To address this possibility, we used two approaches to analyze mitochondrial ROS generation. First, we sorted EpCAM^high^ and EpCAM^low^ cells from treatment naive patient tumor cells and FaDu cells, treated cells with cisplatin and measured the mitochondrial ROS using a fluorescent indicator. ROS activity was decreased at 5, 10, and 20 μM cisplatin concentrations in EpCAM^high^ cells, while increased in EpCAM^low^cells (Fig. 5f) suggesting that therapeutic resistance was partly caused by reducing ROS. Secondly, we knocked down Nrf2 by siNrf2RNA in cisplatin-treated FaDu cells and measured the ROS level. ROS levels steadily increased in both EpCAM^high^ and EpCAM^low^ cells (Fig. 5g).

### Nrf2 inhibition eliminates colony-forming capacity, sphere growth, and invasion capacity in EpCAM^high^ cells

**Fig. 6:**
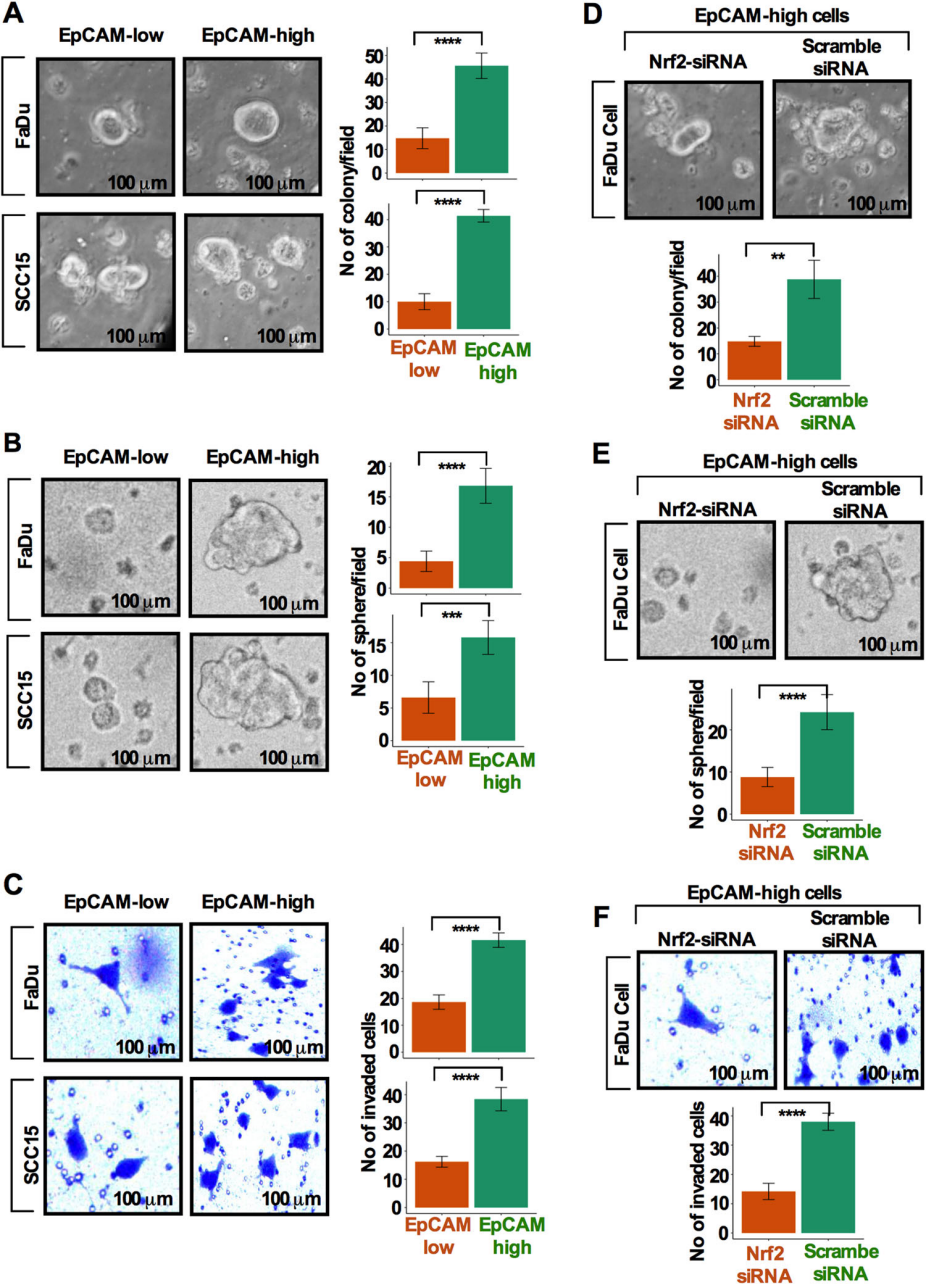
Nrf2 inhibition eliminates colony forming capacity, sphere growth, and invasion capacity in EpCAM^**high**^ cells. **a** Colony-forming assay was carried out and quantified from sorted EpCAM^**high**^ and EpCAM^**low**^ cell populations. **b** Sphere-forming efficiency was determined and quantified using sphere formation assay from EpCAM^**high**^ and EpCAM^**low**^ cell populations. **c** Invasive potential of EpCAM^**high**^ and EpCAM^**low**^ cells was determined and quantified by Transwell invasion assay. *P*-values were calculated using Student’s *t* test. **p < 0.01, ***p < 0.001, ****p < 0.0001. **d**–**f** Numbers of soft agar colonies formed (**d**), sphere formation (**e**), and invasion capacity (**f**) were quantified in siNrf2RNA and si-scramble EpCAM^high^ cells. Scale bar = 100 μm. Values represent mean ± SD from three independent experiments. **p < 0.01; ***p < 0.001; ****p < 0.0001. *P*-values were calculated using Student’s *t* test.

We hypothesize that cells overexpressing EpCAM may acquire higher colony forming capacity, increased sphere growth, and invasion capacity. To test this, FaDu and SCC15 cells were grown in growth factor supplemented CSC medium for 10 days to allow EpCAM enrichment, FACS sorted and quantified for the percent of EpCAM^high^ and EpCAM^low^ cells. Sorted cells were evaluated for the degree of colony-forming capacity, sphere formation, and invasiveness. EpCAM^high^ populations are highly efficient in forming colonies, sphere growth, and invasive capacity compared to EpCAM^low^ cells (Fig. 6a–c). Knockdown of Nrf2 in EpCAM^high^ cells demonstrated reduced colony formation, sphere growth, and invasive capacity as compared to scramble siRNA treated cells (Fig. 6d–f).

### Interleukin-6 and p62 are involved in the activation of the Nrf2 pathway and resistance to cell death in EpCAM^high^ cells

**Fig. 7:**
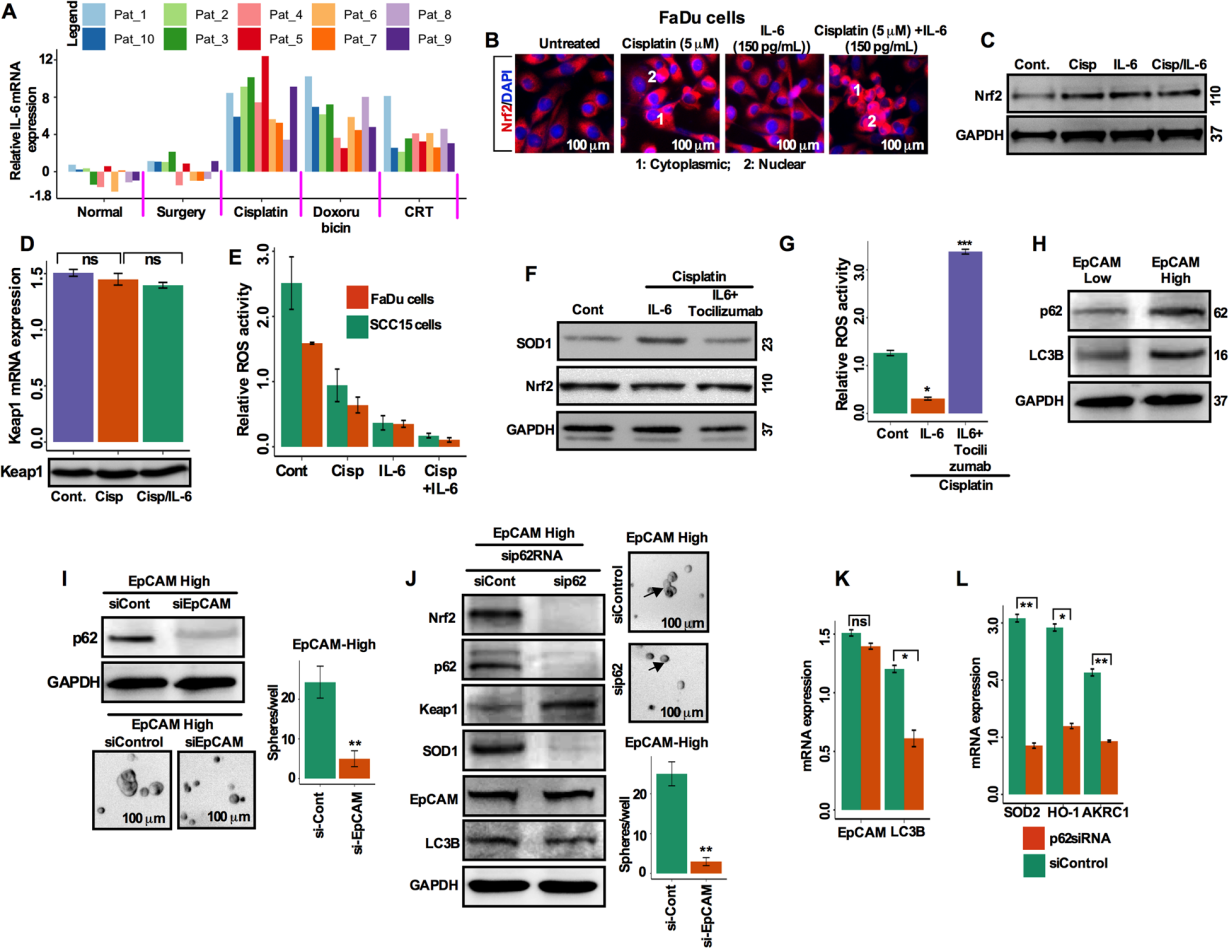
IL-6 (Interleukin-6) and p62 are involved in the activation of the Nrf2 pathway and cell death resistance in EpCAM^**high**^ cells. **a** Real-time PCR analysis of IL-6 expression in HNSCC tumor tissues from matched adjacent normal (*n* = 10), untreated (surgery only; *n* = 10), cisplatin treated (*n* = 10), doxorubicin (*n* = 10), and chemo-radiotherapy (*n* = 10) treated tumor tissues. Transcripts levels were normalized to beta-actin. **b** The immunofluorescence images of cytoplasmic and nuclear Nrf2 in FaDu cells after 5-day cisplatin treatment with or without 150 pg/mL IL-6 (legend: 1-cytoplasmic; and, 2-nuclear Nrf2; Scale bar = 100 μm). **c** Nrf2 protein levels after 5-day post-treatment with cisplatin or combination of cisplatin and 150 pg/mL of IL-6, analyzed by western blotting. **d** Keap1 mRNA and protein expression in FaDu cells 5-day post-treatment with cisplatin alone or with 150 pg/ml of IL-6. **e** ROS level was determined in FaDu and SCC15 cells after treating cells with cisplatin, IL-6 and combination of IL-6 and cisplatin. **f** After 72 h cisplatin treatment with vehicle or 150 pg/mL IL-6 or combination of IL-6 and 30 ng/mL tocilizumab, cell lysates were subjected to western blotting and the levels of SOD1 and Nrf2 proteins were determined. **g** After 72 h cisplatin treatment with vehicle or 150 pg/mL IL-6 or IL-6 with 30 ng/mL tocilizumab, the ROS production was analyzed. **h** p62 and LC3B were measured in EpCAM^**low**^ and EpCAM^**high**^ FaDu cells by western blotting. **i** p62 protein was determined in EpCAM^**low**^ and EpCAM^**high**^ FaDu cells following scrambled siRNA and EpCAM-siRNA transfection. Quantification and representative images of spheres formed by si-scrambled and EpCAM-siRNA transfected EpCAM^**high**^ cells are presented. Scale bar 100 μm. Values represent three separate experiments. **p < 0.01 compared with si-scramble group. **j** EpCAM^high^ cells were transfected with scrambled or p62-siRNA and protein levels of Nrf2, p62, Keap1, SOD1, EpCAM, and LC3B were assessed. Quantification analysis and representative images of spheres formed by si-scrambled and p62-siRNA transfected EpCAM^high^ cells are presented. Scale bar 100 μm. Values represent three separate experiments. **p < 0.01 compared with si-control group. **k**, **l** Transcript levels of EpCAM, LC3B, SOD1, HO-1, and AKRC1 were determined in si-scrambled and p62-siRNA transfected cells by qRT-PCR. Values represent mean ± SD from three separate experiments. *p < 0.05; **p < 0.01. Values compared with si-control group.

Accumulating evidence indicates that both interleukin-6 (IL-6) and the Nrf2-mediated antioxidant pathway contribute to chemotherapeutic resistance in oral squamous cell carcinoma^28,29^. To confirm the role of IL-6 in the activation of Nrf2 in EpCAM^high^ cells, we assessed IL-6 mRNA transcripts from a group of HNSCC patient tumors treated either with chemotherapy (cisplatin; *n* = 10, doxorubicin; *n* = 10) or chemo-radiotherapy (CRT; *n* = 10) or tumors obtained after debulking surgery (*n* = 10) without treatment. IL-6 mRNA was increased in the chemotherapy and chemo-radiotherapy tumors compared with matched adjacent normal and surgery alone tumor (Fig. 7a). To determine the effects of IL-6 on the expression of Nrf2, FaDu cells were treated with either cisplatin (5 μM) or IL-6 (150 pg/mL) alone or in a combination of cisplatin and IL-6 and assessed for Nrf2 expression by immunofluorescence labeling. A detectable increase in Nrf2 expression in the cytoplasm and nucleus was observed in the cisplatin-treated cells (Fig. 7b). Addition of IL-6 significantly increased the cytoplasmic and nuclear Nrf2 expression (Fig. 7b). Western blot analysis showed that IL-6 treatment activated expression of Nrf2 in cisplatin treated cells (Fig. 7c). No changes in Keap1 mRNA and protein expression levels were observed (Fig. 7d). Next, we determined whether IL-6 plays role in preventing or reducing ROS activity under cisplatin and IL-6 treatment conditions. We found that treatment with IL-6 alone reduces ROS generation, while cells treated with cisplatin and IL-6 in combination further reduces the level of ROS (Fig. 7e). Tocilizumab is a humanized anti-human IL-6 receptor monoclonal antibody, which has been shown to controls resistance to radiation by suppressing oxidative stress via Nrf2 pathway^28^. Cisplatin-treated cells undergoing IL-6 and tocilizumab (30 ng/mL) treatment were analyzed by western blot for the expressions of SOD1 and Nrf2. IL-6 alone treatment enhanced SOD1 expression via the Nrf2 pathway, while tocilizumab inhibited the expression (Fig. 7f). In addition, IL-6 treatment significantly reduced the ROS production, while tocilizumab inhibited (Fig. 7g), suggesting that IL-6 is likely involved in the activation of Nrf2 and plays a role in therapeutic resistance by reducing ROS activity.

To analyze the possible involvement of p62 in Nrf2 activation in the chemotherapeutic resistant EpCAM^high^ cells, p62 protein was analyzed by western blotting. p62 protein in the EpCAM^high^ cells was increased, concomitant with an increase in microtubule-associated protein 1A/1B light chain-II (LC3B; Fig. 7h). It appears likely that the increase in p62 is directly related to EpCAM expression (Fig. 7h). Knockdown of EpCAM diminished p62 expression suggesting a correlation between EpCAM and p62 (Fig. 7i). Accordingly, EpCAM silencing in FaDu cells depleted the growth of spheres (Fig. 7i). Silencing of p62 by p62-siRNA revealed the inhibition of Nrf2, p62, and SOD1 (Fig. 7j). Furthermore, p62 knockdown also diminished the efficiency of sphere growth (Fig. 7j). Interestingly, Keap1 expression level increased following p62-mediated silencing (Fig. 7j). The expression of EpCAM remained unchanged after p62-mediated silencing suggesting EpCAM-mediated p62 upregulation in these cells (Fig. 7j, k). The expression level of LC3B was also reduced during p62-mediated silencing (Fig. 7j, k). Silencing of p62 further caused the reduction in expression of Nrf2 target genes, SOD2, HO-1, and AKRC1 (Fig. 7l). All together, these results suggested that Nrf2 activation in EpCAM^high^ CSC-like cells were associated with the increased levels of IL-6 and p62 in HNSCC cells.

## Discussion

In this study, we have shown the role of the Nrf2 pathway activation because the cellular response to electrophilic agents is partially mediated by this pathway and likely plays a significant role in therapeutic resistance through activation of Nrf2, enrichment of CSCs, and lowering of ROS activity. We report that increased Nrf2 activity is associated with the enrichment of CSCs and demonstrated a previously unknown link between EpCAM and the Nrf2 pathway, a leading cause of chemotherapeutic resistance.

Recent studies have highlighted the association between the Nrf2 pathway and CSCs. For example, in neural stem/progenitor cells, Nrf2 overexpression modulated neurosphere formation efficiency as well as neural differentiation^30^. In addition, Nrf2 knockdown in primary human glioblastoma cells decreased the self-renewal capacity of glioma stem cells^31^. As additional evidence, CSCs are highly resistant to conventional chemotherapies, and are considered alternative causes of tumor relapse and aggressiveness. CD44, CD133, CD24, and ALDH activity are frequently used for the detection and isolation of CSCs from tumor tissues and many cancer cell lines. EpCAM has evolved as a potential CSC marker due to its involvement in cell signaling, migration, metastasis, and therapeutic resistance. The link between EpCAM and acquisition of CSC-like properties is supported by EpCAM inhibition. Activation of Wnt/beta-catenin signaling enriched the EpCAM+ cell population, whereas RNA interference-based blockage of EpCAM attenuated CSC activities in cancer cells^32^. These reports highlight a critical role for EpCAM in the development of CSC-like features. In the clinical setting, EpCAM expression is associated with an unfavorable prognosis in breast cancer^33^. Furthermore, low ROS levels are correlated with the maintenance of a subpopulation of drug resistant CSCs within tumors^34^. Since the mechanistic insights into the functions of EpCAM have only been recently explored, the relationship between EpCAM and an association with regulation of the Nrf2 pathway has never been described. Moreover, thus far no studies have explored the association between the Nrf2 pathway and EpCAM expression in the context of CSC-like features and drug resistance. As a molecular mechanism of differential antioxidant capacity and stress resistance of CSCs, we identified a direct association between EpCAM and Nrf2 signaling with respect to drug resistance and enrichment of CSC-like features in HNSCC cells.

Several noteworthy findings have emerged from our study. First, EpCAM was highly expressed in HPV-negative tumors, Nrf2-positive tumors were highly enriched in a EpCAM cell population, and EpCAM was highly expressed in HNSCC tumors compared to normal counterparts. These observations suggest a direct association between EpCAM and the Nrf2 pathway. In concordance, we found that Nrf2 and its target genes were significantly upregulated in the cisplatin-resistant HNSCC tumors compared to cisplatin sensitive tumors. The functional implication is that Nrf2 activation led to the induction of stemness and drug resistance features by overexpressing SOX2 and ABCG5 proteins in EpCAM^high^ cell population, while knockdown of EpCAM by siRNA attenuated the expression of Nrf2, SOD1, and SOX2. Second, CSC-like properties such as robust colony formation efficiency, sphere-forming efficiency, and invasive potential were significantly blocked by knockdown of EpCAM. Furthermore, knockdown of EpCAM sensitized HNSCC cells to the chemotherapeutic drug cisplatin. In addition, Nrf2 knockdown in EpCAM^high^ cells resulted in significant increase in ROS activity, loss of colony formation, sphere growth, and invasiveness as well as cisplatin-mediated resistance, stemness, and CSC enrichment. These results provide a new mechanistic molecular basis for the modulation of CSC characteristics and interplay between EpCAM and the Nrf2 signaling pathway and resulting therapeutic resistance.

Our findings support the association of EpCAM and the Nrf2 signaling pathway, which in combination may play a crucial role in CSC-like characteristics and thereby limiting the success of therapeutic outcomes. Notably, it was found that breast cancer cells transform to CSC-like cells after prolonged incubation with anticancer drugs with induction of Nrf2 target genes^35^. In breast and colon cancers, elevated Nrf2 levels induce the drug efflux transporters, chemoresistance, and spheroid growth^20,36^. Furthermore, Nrf2 inhibition reversing the resistance of cisplatin-resistant HNSCC has recently been reported^21^. A recent study has shown the involvement of the CD44-p62 pathway in inducing the Nrf2 pathway in CD44-positive cells^37^. All these reports suggest the active involvement of Nrf2 in acquisition of CSC-like features leading to stress-associated drug resistance and therapeutic failure.

Lastly, from a mechanistic point of view, our results demonstrated that modulation of IL-6 and autophagy-associated p62 contributed to the activation of Nrf2 in EpCAM^high^ CSC-like cells. Chemotherapeutic treatment is known to significantly contribute to ROS generation in various cancers and contribute to the oxidative stress response^38^. On the other hand, IL-6 protects cancer cells from chemotherapy-induced oxidative DNA damage^28^ and promotes DNA repair in CD133-positive cancer stem cell-like cells^39^. Very few studies have shown how IL-6 might activate the Nrf2 signaling pathway upon chemotherapy. In this study, we provide evidence that IL-6 may contribute to activation of the Nrf2 pathway, resulting in chemotherapeutic resistance by reducing ROS activity in HNSCC cells. In addition to IL-6, p62 contributes to Nrf2 pathway activation. Several prominent studies reported the involvement of p62 in CSC maintenance and CSC-related resistance to therapy. Activation of autophagy results in inhibition of apoptosis and high p62 expression has been associated with advanced clinical stage and correlated with high invasive and metastatic efficiency in endometrial cancer^40^. Furthermore, high levels of p62 level were found in spheroids, CD44-high/CD44-low cells, and ALDH-positive subpopulations in breast CSC-like cells^37^. These reports highlight that p62 plays crucial roles in Nrf2-mediated stress resistance. However, as knowledge is limited about the association and mechanism of p62 elevation in CSC-rich cell population, we explored this issue particularly in the EpCAM^high^ population. We have shown that, EpCAM^high^ cells showed increased p62 and LC3B-II expression when compared to EpCAM^low^ cells, suggesting the activation of autophagy in EpCAM^high^ cells. These observations suggest that activation of IL-6 and p62 in CSC-like EpCAM^high^ cells might be an important regulatory axis for CSC-like cell survival and therapeutic resistance in HNSCC.

In conclusion, our data support the contention that Nrf2 activation is an important molecular mechanism in cancer coupled with the functional involvement of IL-6/p62 in EpCAM^high^ HNSCC. Therefore, it is highly possible that, activated Nrf2 in EpCAM^high^ cells contributed to the acquisition of aggressive CSC-features and to leading to chemotherapeutic resistance. On the basis of our observations we propose that the EpCAM-Nrf2 pathway might be an interesting and potential therapeutic target for the elimination of the stress-mediated drug resistance and survival of EpCAM^high^ subpopulation. Although our limited in vitro studies using two cell lines and patient material are convincing, they do not yet allow us to draw definitive conclusions. Future studies should include HNSCC stem cell xenograft models to dynamically test the EpCAM-Nrf2 drug resistance paradigm.

## Materials and methods

### Cell culture and patient samples

SCC15 and FaDu cells were purchased from American Type Cell Culture (ATCC). Cells were cultured in DMEM supplemented with 10% FBS and 1% penicillin and streptomycin cocktail. Formalin-fixed paraffin-embedded tissues from 100 HNSCC patients treated in Chittagong Medical College Hospital (CMCH), Chittagong were included in this study after obtaining full patient consent. The study protocol for collection and use of patient tumor tissues and use of clinical information was approved by the central Bangladesh Medical Research Council (BMRC) ethics committee of Bangladesh (approval ID no: 052(1) 04 06 2014). After obtaining patients’ informed consent and following local and international regulations, HNSCC tumors were obtained from all consented patients at the time of surgery. Collected tumors were first minced and enzymatically dissociated with 2 mg/mL of dispase (Roche, CA, USA) and then incubated with 0.25% Trypsin-EDTA, passed through a 21-gauge syringe and filtered through a 23-μm cell filter (Merck Millipore). Cells were either directly cultured in supplemented CSC medium or cryopreserved in 80% fetal bovine serum (FBS) and 20% dimethylsulfoxide (DMSO) until further use.

### AlamarBlue cytotoxicity and proliferation assay

Cells were seeded (5000 cells/well) in a 96-well plate in complete medium. Cells were treated with an increasing concentration of cisplatin (0–80 μM) for 72 h. Cell viability was assessed by AlamarBlue (Thermo Fisher, MA, USA) assay using manufacturer’s instructions. AlamarBlue was added (10% of total volume) and incubated for 4 h in an incubator and fluorescence was measured using the SPECTRAmax Gemini Spectrophotometer (540 nm excitation and 590 nm emission). DRC (Dose-response curve) package was used to generate dose-response curves using R-Statistical software. Inhibitory EC50 concentration values were calculated (DRC package) from the results of varying cisplatin concentrations, in triplicate and from three independent experiments.

### Nrf2, p62, and EpCAM siRNA transfection assay

Nrf2 (siGENOME D-003755-01), EpCAM (siGENOME D-004568-04), and p62 (siGENOME D-010230-02) specific siRNA and non-targeting scramble siRNA sequence (siGENOME D-001210-01) were obtained from Dharmacon. Transfection were performed in 50% confluent cell cultures using Lipofectamine 3000 (Thermo Fisher, MA, USA) and cultured in reduced serum medium OPTI-MEM following manufacturer’s instructions. The specific siRNAs were transfected into SCC15 and FaDu cells done in triplicate.

### ARE luciferase assay

HNSCC cells (2 × 10^5^ cells/well) were plated in six-well plates in complete medium containing 10% FBS. Cells were first transfected with ARE-luciferase plasmid (20 μL/well) and Lipofectamine 3000 transfection reagents (Thermo Fisher, MA, USA) according to manufacturer’s instruction. After 24 h, cells were treated either with siEpCAM or scrambled siRNA for 48 h. Luciferase activity was detected using the dual luciferase assay kit (Promega, WI, USA). Relative luciferase activity was calculated according to the relative light unit (RLU) of the firefly luciferase divided by the RLU of the renilla luciferase.

### Real-time quantitative polymerase chain reaction

Total RNA was extracted from fresh tumor tissues, SCC15 and FaDu cells using RNAeasy Kit (Qiagen, MD, USA) and reversed transcribed. Total RNA was isolated from formalin-fixed, paraffin-embedded tumor tissue sections using RNAeasy FFPE kit (Qiagen, MD, USA) and reverse transcribed. SYBR-Green-1 based RT-PCR amplification was performed in triplicates on the LightCycler-480 (Roche, CA, USA). The primers list is shown in Supplementary Table S1. The relative expression of each gene was analyzed by comparing its expression to that of GAPDH.

### Western blotting

Lysed protein was transferred to PVDF membrane and primary antibodies were added to PVDF membranes in 5% non-fat dry milk in TBS-Tween-20 buffer. Primary antibodies are: EpCAM (Cell Signaling Technology), Sox2 (Cell Signaling Technology, MA, USA), ABCG5 (Thermo Fisher), Nrf2 (AbCam, MA, USA), SOD1 (AbCam, MA, USA), p62 (AbCam, MA, USA), LC3B-II (AbCam, MA, USA), and GAPDH (Santa Cruz, CA, USA). HRP-conjugated anti-mouse or anti-rabbit secondary antibodies were used for the detection.

### Immunohistochemistry and immunofluorescence

The following primary antibodies against Nrf2 (AbCam, MA, USA), CD44 (AbCam, MA, USA), CD133 (Cell Signaling Technology, MA, USA), CD49f (Thermo Fisher, MA, USA), Sox2 (Cell Signaling Technology, MA, USA), and EpCAM (Cell Signaling Technology, MA < USA) were applied on deparaffinised 5-μm-thick formalin-fixed tissue sections for overnight. A horseradish peroxidase (HRP)-conjugated secondary antibody was used for the detection. For Nrf2 detection in cells, only nuclear immunostaining was included in this study because only transcriptionally active Nrf2 resides in the nucleus. The expression intensity was divided into four groups based on the percentage of cells showing positive nuclear staining for Nrf2 and Sox2, or positive cell surface staining for CD44, CD133, CD49f, and EpCAM. 0 no staining; 1+ <10–25%; 2 + 26–50%; 3 + 51–75%; and 4 + >75% of positive cells. For EpCAM immunofluorescence staining FITC-conjugated secondary antibody was used and analyzed under a fluorescence microscope.

### Colony-forming assay

Cells were resuspended in 40% methylcellulose with complete medium and plated in 35 mm tissue culture plates (Nalgene, NY, USA) in triplicate and incubated in 5% CO_2_ at 37° C for 2 weeks and the numbers of colonies counted under phase contrast microscopy. Clonogenic efficiency was determined as the average number of colonies per dish for each group. For clonogenic efficiency of cells transfected with siRNA, the same procedures were followed except cells were prior treated with either siRNA or scrambled siRNA.

### Sphere-forming assay

siRNA treated and scrambled siRNA cells were cultured in six-well ultra-low attachment plates at a density of 1000 cells/well in growth factor supplemented CSC medium. The number and size of the spheres were monitored and recorded every 3 days. Sphere-forming efficiency was calculated as the number of actual spheres/number of cells plated × 100.

### Invasion assay

Harvested siRNA treated or scrambled-siRNA treated cells (50,000 cells/well) were seeded in Matrigel coated upper chambers in low serum medium. After 24 h of incubation, upper chamber cells were wiped off and remaining cells stained with 1% crystal violet for 30 min at room temperature. After washing the cells 3x with PBS, the numbers of attached invaded cells were counted under phase contrast microscope. The experiments were repeated in triplicate.

### Fluorescence-activated cell sorting and flow cytometry

Patient tumor cells, SCC15 and FaDu cells were stained on ice and then isolated by FACS using BD FACSAria flow cytometer and analyzed by BD FACSDiva. For isolation of EpCAM cell subpopulations, cells were sequentially stained with EpCAM-APC conjugated antibody. Isolated EpCAM^high^ and EpCAM^low^ cells were washed and cultured in 3D in vitro sphere culture supplemented CSC medium. Cells were treated with either scrambled siRNA or siEpCAM when necessary.

### Measurement of ROS by DCFDA (2′-7′- dichlorodihydrofluorescein diacetate) assay

ROS levels were measured after dissociating cells from spheres and incubation in DCFDA (Sigma Aldrich, SC, USA). Unless otherwise indicated, cells were treated with IL-6 (150 pg/mL) and tocilizumab (30 ng/mL, Roche, MA, USA) followed by cisplatin for 72 h, trypsinized and allowed to form spheres in CSC medium and incubated for 7 days. Spheres were dissociated with 0.05% trypsin-EDTA at 37 °C for 3–5 minutes and centrifuged for 5 min. Cells were then resuspended with medium, 20 μM DCFDA added and incubated at 37 °C for 90 min in the dark. DCFDA fluorescence intensity was detected by flow cytometry, using FITC channel on BD FACSAria flow cytometer (BD BioScience, CA, USA). Cell lysates were collected for protein analysis.

### Statistical analysis

Experiments were repeated three times and results were presented as mean ± SD. For independent data with two specimens, a two-tailed *t*-test for equal variance, or one-way ANOVA Tukey post-hoc comparison for three or more groups were applied. Comparison between groups were evaluated using non-parametric Mann–Whitney U test. For all statistical analysis, we used “R” statistical software (version 3.6.1), and for graphs “ggplot2” packages in R. Kaplan–Meier survival curves were generated and analyzed using R package “survival” and “survminer”. The significance was calculated using log-rank and Mantel–Cox test. Dose response was analyzed using a DRC package (dose response curve) in R statistical software.

## Supplementary information

Supplementary Figure Legend

Supplementary Figure S1

Supplementary Figure S2

Supplementary Table S1

## Data Availability

All dataset supporting the conclusions of this manuscript is included within the article
