# Supplementary material for: Chemotherapeutic resistance of head and neck squamous cell carcinoma is mediated by EpCAM induction driven by IL-6/p62 associated Nrf2-antioxidant pathway activation
Source: Cell Death Dis. 2020 Aug 20;11(8):663. doi: 10.1038/s41419-020-02907-x (PMC7438524; doi:10.1038/s41419-020-02907-x)
Supplement: Supplementary file 1 — Supplementary Figure Legend [file 41419_2020_2907_MOESM1_ESM.docx]

**Supplementary Figure legend**

**Supplementary Figure S1:** **A.** Cell viability of parental FaDu and FaDuRes was determined following cisplatin treatment for period of 48 h. **B.** Confirmation of the overexpression of EpCAM following cisplatin treatment for 48 h.

**Supplementary Figure S2:** EpCAM specific siRNA (si-EpCAM) was transfected into EpCAM-high cells and knock down efficiency of EpCAM gene was determined by Western blot. Non-specific scrambled siRNA (sc) was used as negative control.
