# Supplementary figures and images for: Chemotherapeutic resistance of head and neck squamous cell carcinoma is mediated by EpCAM induction driven by IL-6/p62 associated Nrf2-antioxidant pathway activation
Source: Cell Death Dis. 2020 Aug 20;11(8):663. doi: 10.1038/s41419-020-02907-x (PMC7438524; doi:10.1038/s41419-020-02907-x)

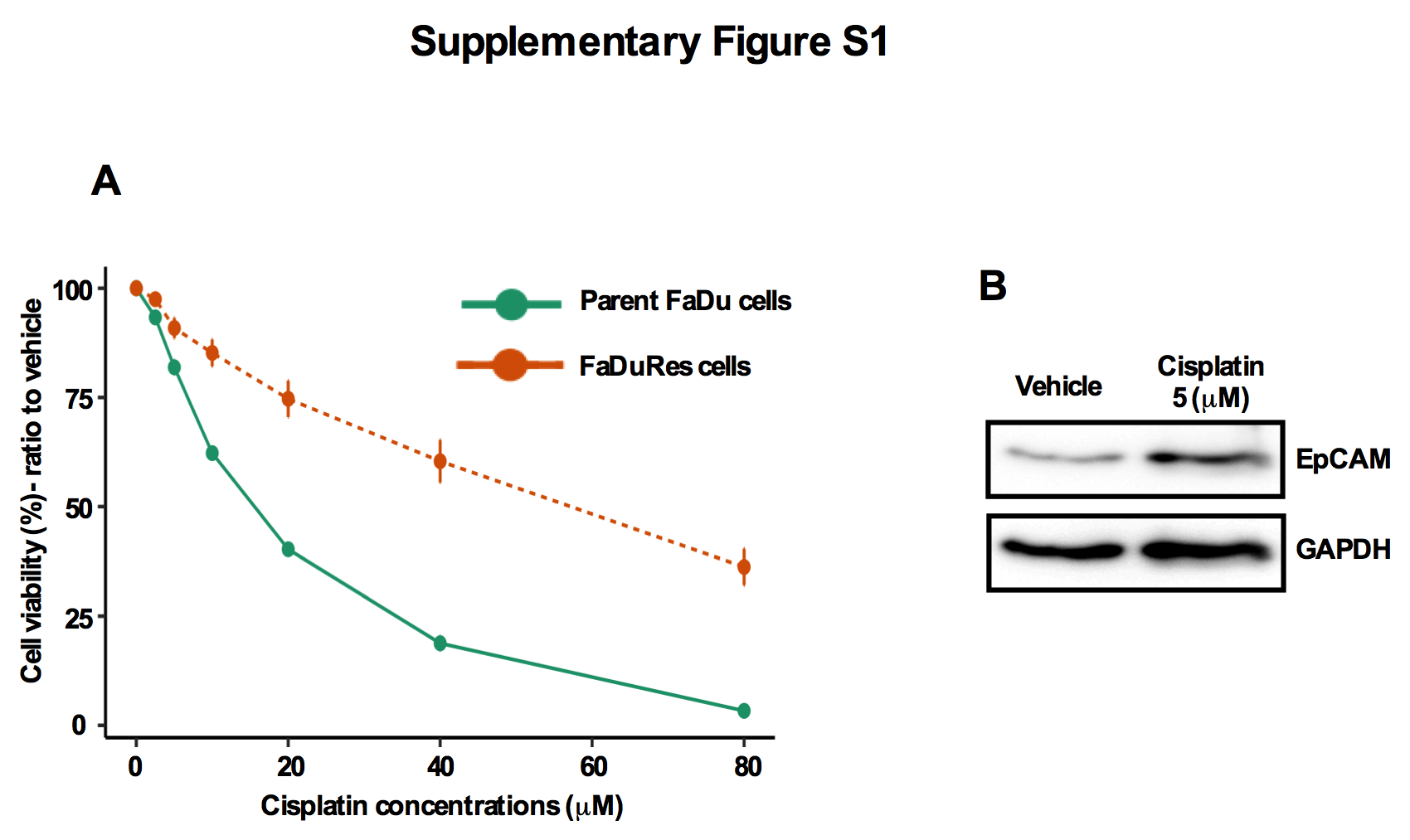

Supplement: Supplementary file 2 — Supplementary Figure S1 [file 41419_2020_2907_MOESM2_ESM.tif]

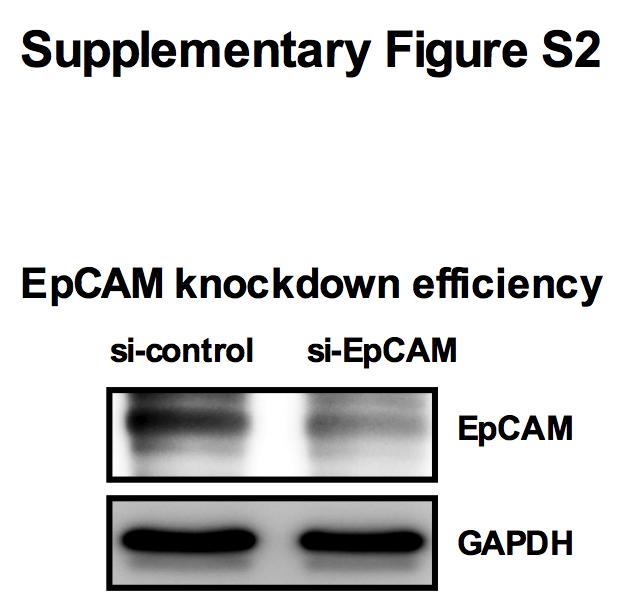

Supplement: Supplementary file 3 — Supplementary Figure S2 [file 41419_2020_2907_MOESM3_ESM.tif]
