# Supplementary material for: Chemotherapeutic resistance of head and neck squamous cell carcinoma is mediated by EpCAM induction driven by IL-6/p62 associated Nrf2-antioxidant pathway activation
Source: Cell Death Dis. 2020 Aug 20;11(8):663. doi: 10.1038/s41419-020-02907-x (PMC7438524; doi:10.1038/s41419-020-02907-x)
Supplement: Supplementary file 4 — Supplementary Table S1 [file 41419_2020_2907_MOESM4_ESM.docx]

| ***Gene names*** | ***Left primers*** | ***Right primers*** |
| --- | --- | --- |
| ***Nrf2*** | GCGACGGAAAGAGTATGAGC | GTTGGCAGATCCACTGGTTT |
| ***Keap1*** | CCTTCAGCTACACCCTGGAG | CATGACCTTGGGGTGGATAC |
| ***EpCAM*** | GCTGGTGTGTGAACACTGCT | ACGCGTTGTGATCTCCTTCT |
| ***p62*** | CAGTGGCTCACCCCTGTAAT | ACACCTCCTGGGTTCAAGTG |
| ***SOD1*** | AGGGCATCATCAATTTCGAG | ACATTGCCCAAGTCTCCAAC |
| ***SLC3A1*** | GGGAACAGCGTGTATGAGGT | GGAGTTCCAGGGAGTGTGAA |
| ***AKR1C1*** | ACCACAGCTGGTGCTCTTTT | TGACTGGTCAGGGTGTGGTA |
| ***MRP1*** | AAGAAAACAGGGAAGCAGCA | GCTCTCTGGGTTTGAAGTCG |
| ***MRP2*** | TGCTTCCTGGGGATAATCAG | CACGGATAACTGGCAAACCT |
| ***GCLM*** | TGGAAATGCCCAACATTTTT | TGAGGGTGCAGGTAGGAGAT |
| ***POR*** | TCTACGACATCGTGGCTGAG | CCAAACACACCCAGGAGACT |
| ***CRYZ*** | CAGCATGCCAAATTGCTAGA | CTCGTCCTCCATGTGACAGA |
| ***TXN*** | CTGCTTTTCAGGAAGCCTTG | TTGGCTCCAGAAAATTCACC |
| ***GCLC*** | AGAGAAGGGGGAAAGGACAA | GTGAACCCAGGACAGCCTAA |
| ***GPX2*** | CAAGCGCCTCCTTAAAGTTG | GAGGGTTGGGAGAGGAAAAG |
| ***HO-1*** | TCCGATGGGTCCTTACACTC | TAAGGAAGCCAGCCAAGAGA |
| ***NQO1*** | TTACTATGGGATGGGGTCCA | TCTCCCATTTTTCAGGCAAC |
| ***SOD2*** | GCCATTGCTTTTGGTGTTTT | AAATGGTGCTGGGAAAACTG |
| ***LC3B*** | CCACACCCAAAGTCCTCACT | CACTGCTGCTTTCCGTAACA |
| ***IL-6*** | TACCCCCAGGAGAAGATTCC | TTTTCTGCCAGTGCCTCTTT |

**Supplementary Table S1: List of primers for qRT-PCR**
